# Supplementary material for: Motor representations underlie the reading of unfamiliar letter combinations
Source: Sci Rep. 2020 Mar 2;10:3828. doi: 10.1038/s41598-020-59199-6 (PMC7052247; doi:10.1038/s41598-020-59199-6)
Supplement: Supplementary file 1 — Supplementary Information. [file 41598_2020_59199_MOESM1_ESM.pdf]

Supplementary Material

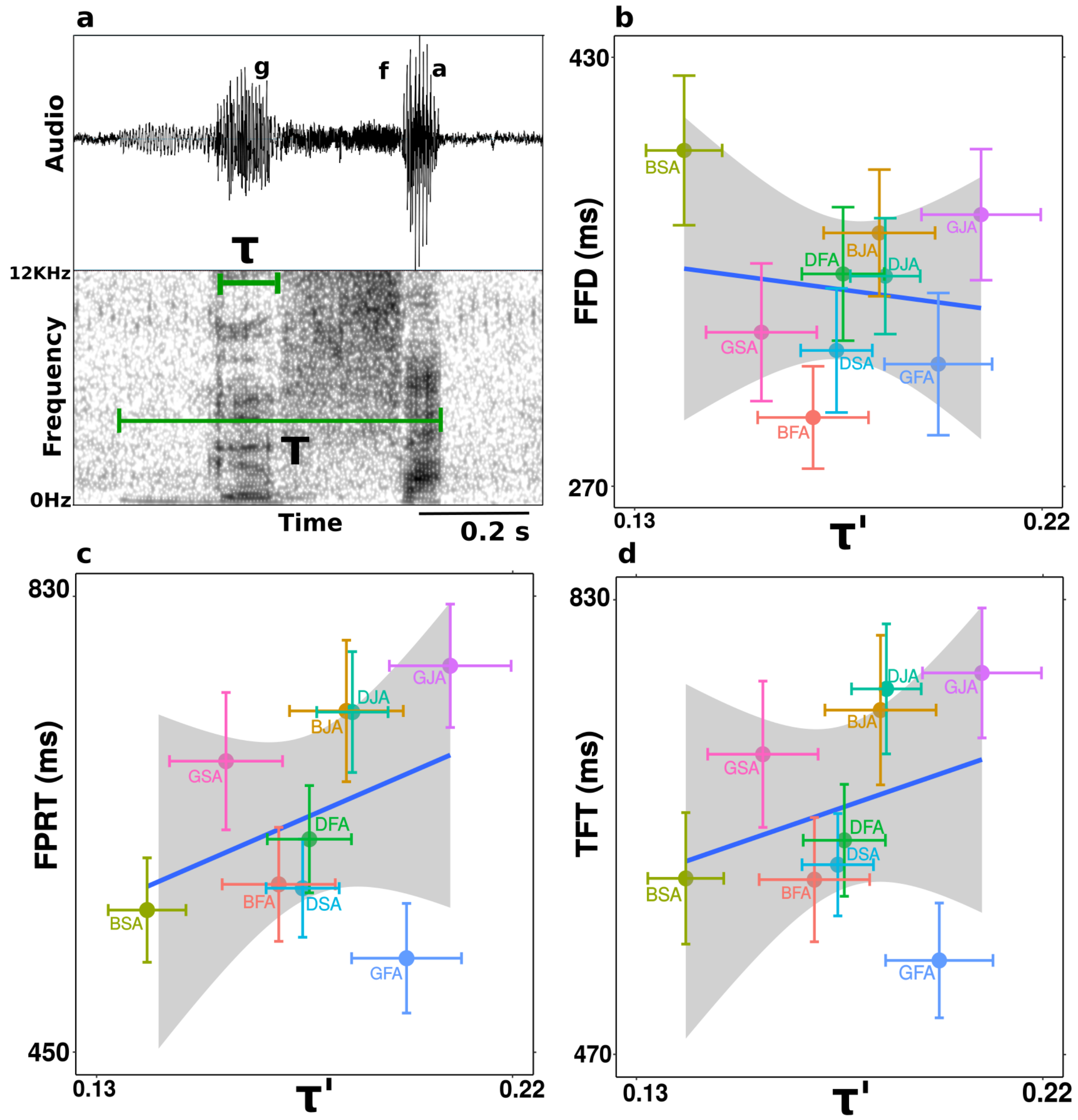

Figure S1 | Voiced plosive-fricative combinations. a. Audio and spectrogram example from /GFA/ pronunciation. Transitions are characterized by the interval that goes from releasing the occlusion of the plosive to the formation of a constriction to generate the fricative. Spectrally, the transition is described by the voiced sound structure produced after the plosive, and before the formation of the purely noisy fricative spot. **b - d**. Linear regression test was conducted for the variables TFT, FPRT and FFD with consonantal transition as the independent variable. Means and standard errors per CCV were considered for the regression as data points come from different block. Although a positive tendency is observed, any of the variables hold a statistically significant relation with τ' **:** FFD ($t(7)=-0.16, p=0.87$) FPRT ($t(7)=1.07, p=0.32$) and TFT ($t(7)=0.76, p=0.47$)

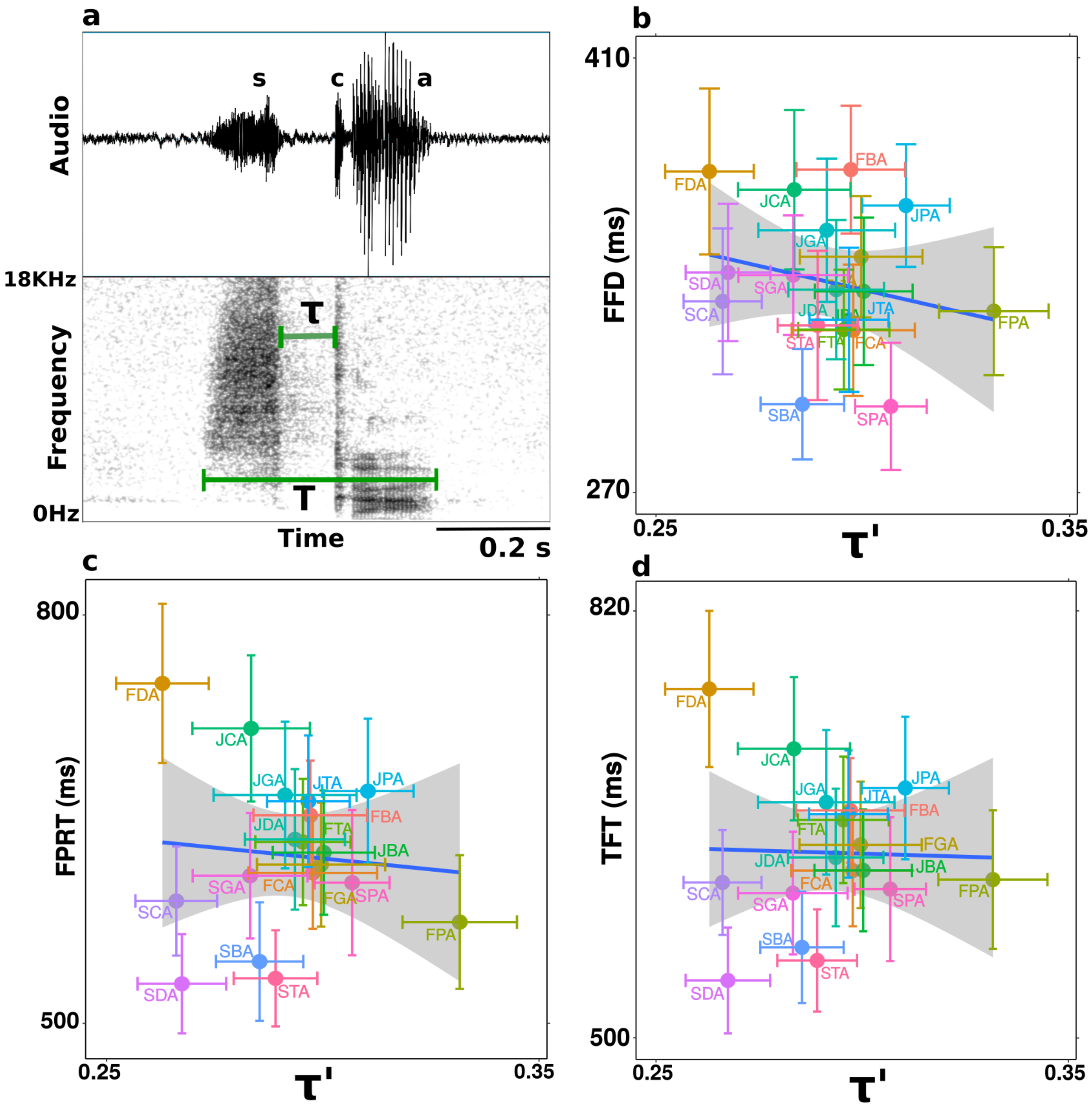

Figure S2 | Fricative–plosive combinations. a. Audio and spectrogram example from /SCA/ pronunciation. During these transitions, the vocal tract evolves from a constriction at one point to an occlusion at another one. Transitions are spectrally defined as the interval between the abrupt end of the fricative and the release of the plosive into the vowel [a]. **b - d.** Linear regression test was conducted for the variables TFT, FPRT and FFD with consonantal transition as the independent variable. Means and standard errors per CCV were considered for the regression as data points come from different block. Any of the variables hold a statistically significant relation with τ' : FFD ($t(16)=-0.73, p=0.48$) FPRT ($t(16)=0.06, p=0.95$) and TFT ($t(16)=0.27, p=0.79$)

**Voiced Plosive - Fricative**	**Intra-word frequency**
Vocal onset delay Δ	F(2, 275) = 2.33, *p* = 0.10
Total fixation time TFT	F(2, 883) = 0.18, *p* = 0.83
First pass FPRT	F(2, 883) = 0.23, *p* = 0.79
First fixation FFD	**F(2, 883) = 3.38, *p* = 0.035**
Consonantal transition τ'	**F(2, 275) = 6.45, *p* = 1.8 10^{-3}**
**Fricative - Plosive**	
Vocal onset delay Δ	F(2, 592) = 0.19 , *p* = 0.66
Total fixation time TFT	**F(2, 1762) = 6.08, *p* = 0.014**
First pass FPRT	**F(2, 1762) = 7.99, *p* = 4.8 10^{-3}**
First fixation FFD	F(2, 1762) = 2.36, *p* =0.13
Consonantal transition τ'	**F(2, 592) = 4.65, *p* = 0.032**

Table S1. Effects of frequency on phonatory and ocular variables during reading CCVs in the fricative - plosive cluster and voiced plosive – fricative cluster. An ANOVA test revealed significant effect of intra-word frequency on of the variables. Significance level was set to $\alpha = 0.05$ to account for unique comparisons.
